# Electroporation Therapy in Soft Tissue Sarcoma: A Potentially Effective Novel Treatment

**DOI:** 10.1155/SRCM/2006/85234

**Published:** 2006

**Authors:** Remco de Bree, Bernard M. Tijink, Cees J. van Groeningen, C. René Leemans

**Affiliations:** ^1^Department of Otolaryngology/Head and Neck Surgery, VU University Medical Center (VUmc), De Boelelaan 1117, 1081 HV Amsterdam, The Netherlands; ^2^Department of Medical Oncology, VU University Medical Center (VUmc), De Boelelaan 1117, 1081 HV Amsterdam, The Netherlands

## Abstract

*Purpose*. Examination of the potential of electroporation therapy (EPT) in a patient with metastatic soft tissue sarcoma. *Patient*. A 24-year-old male who underwent extensive resection and postoperative radiotherapy for a malignant peripheral nerve sheath tumor in the right infratemporal fossa with intracranial extension and invasion of the maxillary sinus and mandible had a recurrence in the scar of his craniotomy for which he was initially treated with doxorubicin. After discontinuation of doxorubicin he developed a metastatic mass at the same site for which he was treated with electroporation therapy. *Method*. The subcutaneous metastasis was infiltrated with bleomycin and electroporated. *Results*. Gradually the tumor became increasingly necrotic and demarcated from surrounding tissue. After 10 weeks no tumor was seen anymore. The wound healed secondarily. *Discussion*. Intralesional bleomycin followed by EPT is potentially effective, well tolerated, and easy to perform in well accessible soft tissue sarcoma sites.

## INTRODUCTION

Sarcomas in the head and neck area are rare. The management of soft tissue sarcomas in the head and neck is primarily surgical. However, the critical anatomy of the head and neck limits the capacity to obtain wide surgical margins. Postoperative radiotherapy improves the local control rate [1, 2]. Despite this combination of treatment modalities, a higher local recurrence rate and a worse disease-specific survival of head and neck sarcoma patients are found as compared to sarcomas arising at other sites [3–5]. The results of adjuvant chemotherapy in treatment of head and neck soft tissue sarcomas have been disappointing [6].

**Figure 1:**
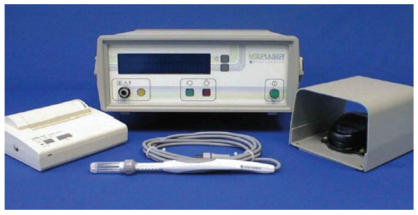
MedPulser electroporation device from Genetronics Biomedical Corporation (San Diego, California, USA). Permission granted by Genetronics Inc Pulse generator, foot switch, and applicator.

Electroporation therapy (EPT) is a novel local treatment modality that uses brief, high-intensity, pulsed electrical currents to enhance the uptake of cytotoxic drugs, vaccines, and genes into cells by producing a transient increase in cell wall permeability [7–9]. The technique is potentially useful in primary and secondary tumors of different tumor types. EPT involves the use of a specially developed delivery device, the MedPulser (Genetronics Biomedical Corporation, San Diego, California, USA), which consists of a circular array of electrode needles [10]. This needle array is inserted directly into the lesion (Figure 1). Experience has been achieved with various tumor types, but there is yet no report on soft tissue sarcoma.

## CASE REPORT

A 24-year-old male was referred to our institute because of hearing loss and progressive pain complaints in his face on the right side since one month. Physical examination showed a glue ear on the right side and mild trismus. No other abnormalities were found, especially no mucosal lesions or swelling in the neck.

**Figure 2:**
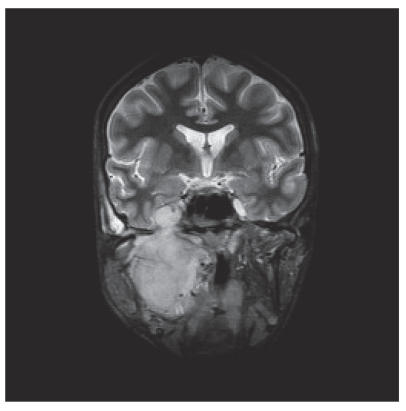
MRI (coronal view) showed the tumor in the right infratemporal fossa with intracranial extension and invasion of the maxillary sinus and mandible.

CT and MRI showed a large tumor in the right infratemporal fossa with intracranial extension through the foramen ovale to the cavernous sinus and growth in the posterior wall of the maxillary sinus and destruction of the ascending part of the mandible (Figure 2).

A transantral biopsy through the maxillary sinus showed on histopathological examination a malignant peripheral nerve sheath tumor. CT-scans of the chest and abdomen and bone scintigraphy did not show signs of distant metastases.

**Figure 3:**
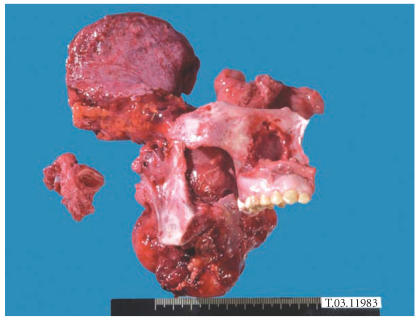
Surgical specimen including maxillectomy, segmental mandibula resection, and selective neck dissection.

He underwent a resection consisting of craniotomy, parotidectomy, maxillectomy, and segmental mandibula resection with selective neck dissection of level I-III. The defect was reconstructed with a free vascularized iliac crest flap. His postoperative course was uneventful. Histopathological examination of the surgical specimen (Figure 3) revealed positive surgical margins. No lymph node metastases were found in the neck dissection specimen. He received postoperative radiotherapy to a total dose of 60 Gy in 30 fractions.

**Figure 4:**
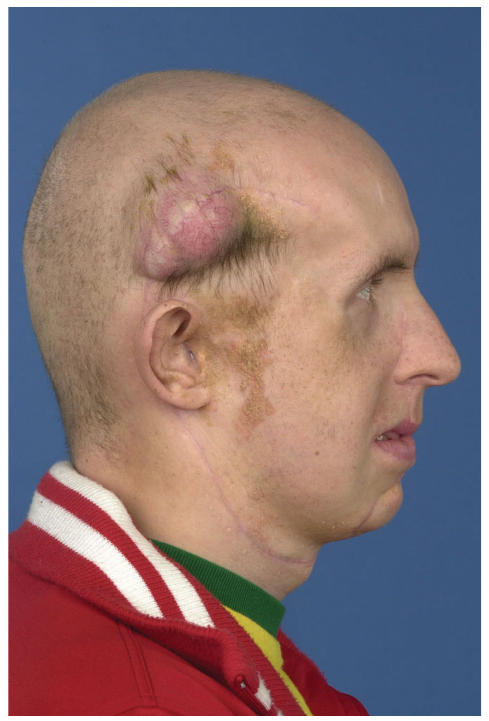
Subcutaneous metastasis in the scar of the craniotomy 5 months after initial treatment.

**Figure 5:**
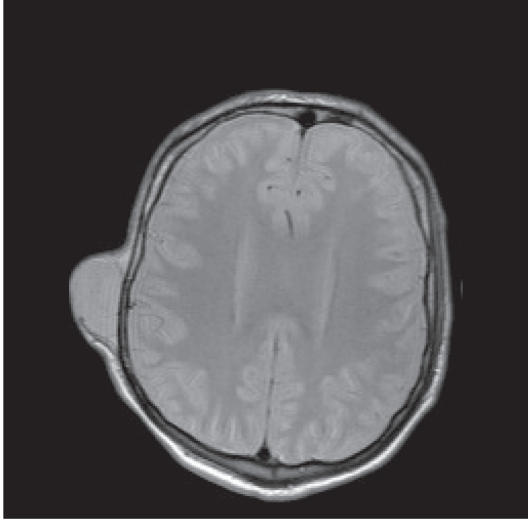
MRI (axial view) showed the subcutaneous temporal metastasis with intact underlying bone.

Unfortunately, 5 months post-operative he developed a subcutaneous metastasis in the temporal scar of the craniotomy (Figure 4). An MRI showed that the underlying bone was intact (Figure 5). Metastases were also present in the neck on the right-hand side. He was treated with 8 cycles of doxorubicin. There was a complete response of both lesions. After one month he developed a recurrence on the same place of his scalp again. An MRI showed no signs of recurrence in the neck.

**Figure 6:**
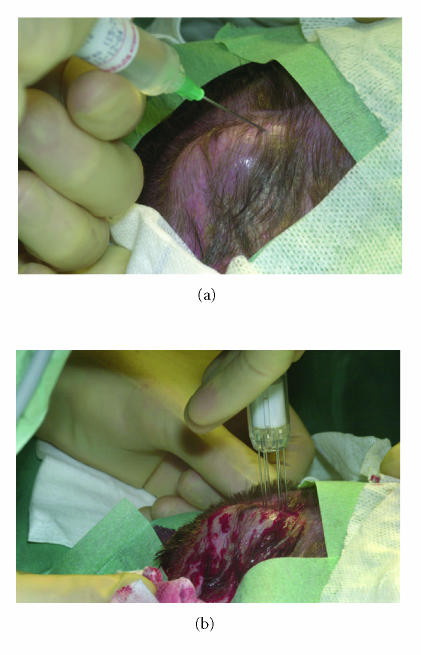
(a) Intralesional injection of bleomycin. (b) Electroporation therapy.

**Figure 7:**
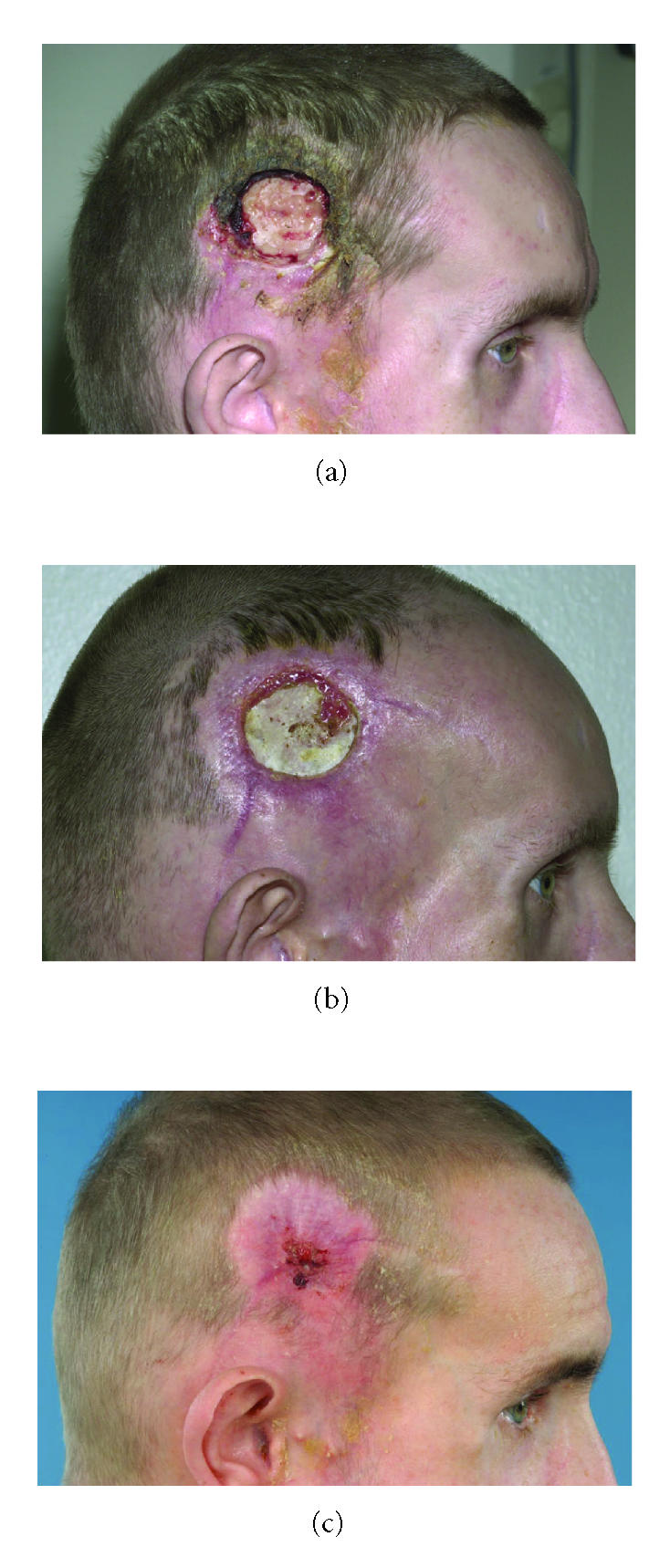
Lesion after 5 weeks (a), 10 weeks (b), and 12 months (c).

Since no accepted standard treatment was left, we decided to propose an experimental approach in the form of intratumoral injection of bleomycine with electroporation therapy. After informed consent was obtained, the lesion of 50 × 35 mm was uniformly infiltrated with 60 USP-E bleomycin (4 USP-E/mL) under general anesthesia. After five minutes the tumor and a safe margin of 0.5 cm were electroporated using a six-needle array applicator connected to the MedPulser system (Genetronics Inc, San Diego, California), which transforms sinusoidal electrical energy pattern [10]. Tumor and margin were treated with 63 overlapping applicator placements (Figure 6). Gradually the tumor became increasingly necrotic and demarcated from the surrounding tissue. The wound was covered by hemorrhagic crusts. He had no pain complaints. After 10 weeks no tumor was seen anymore. Secondary healing started from the margins of the wound (Figure 7). Two months after EPT he developed a recurrence in his neck, for which he was treated with ifosfamide. This wound healing was delayed due to his chemotherapy courses. He is presently (17 months after electroporation) without disease on his scalp and is doing reasonably well.

## DISCUSSION

Bleomycin, a cytotoxic agent derived from actinomycetes, is a purified glycopeptide that inhibits DNA synthesis by inducing single-and double-stranded DNA breaks. It is hydrophilic and does not cross the cell membranes readily [11, 12]. EPT temporarily increases the permeability of cell membranes by creating transient pores. These pores permit direct diffusion of drugs into the cells bounded by these cell membranes, achieving higher intracellular concentrations than can be obtained by intralesional injection alone. Not only does this permit the uptake of substances into the cytoplasm, but it also enhances the uptake in the nucleus [7]. EPT dramatically enhances the uptake of bleomycin into cells. Bleomycin has been demonstrated *in vitro* to exhibit the greatest increase in cytotoxicity after EPT (a 700-fold increase) [11]. These results have been replicated *in vivo* using tumor cell lines, including melanoma, sarcoma, and carcinoma cell lines implanted subcutaneously in nude mice [13–15]. These models demonstrated the superiority of bleomycin in EPT over the other drugs tested with EPT [10, 16–18].

The electric field for EPT is generated by the MedPulser system. Optimization of pore formation has been achieved using a circular six-needle array applicator. Switching polarity of paired needles with a second pulsing and rotating the field by 60 degrees for three cycles results in a circle of positive and negative field pulses that maximize pore formation in tumor cells within the fields. Several types of probes with different sizes and shapes have been developed for application in different kinds of tumors in different parts of the body [19].

In some clinical studies it has been shown that in cutaneous lesions the response rate of bleomycin and EPT is significantly higher than intralesional bleomycin alone, while EPT alone showed no clinical response [16, 20]. It has been shown that EPT with low local concentrations of bleomycin after intravenous administration has substantially less effect than EPT following intratumoral injection of bleomycin [18].

The majority of clinical papers on EPT report on successful treatment of cutaneous malignancies, including squamous cell carcinomas, basal cell carcinomas, and melanomas [18, 21, 22]. In melanoma patients complete response rates of 72% to 89% and partial response rates of 5% to 17% have been reported [16, 20, 22]. The clinical use of bleomycin and EPT to treat mucosal squamous cell carcinoma of the head and neck has been reported [23]. EPT was also used successfully in patients with chondrosarcoma and Kaposi's sarcoma [22, 24]. This is the first clinical report on a successful treatment of soft tissue sarcoma.

Although surgery with postoperative radiotherapy on indication is likely to remain the treatment of choice for primary resectable sarcomas, EPT after intralesional injection with bleomycin may represent an alternative to surgical resection of recurrent disease. In certain settings, like in our patient, EPT may be advantageous in terms of local tissue preservation, improved quality of life and costs. Other potential advantages are outpatient treatment feasibility and response in settings in which conventional therapy has failed. EPT could be particularly useful in previously operated fields, for controlling symptomatic localized disease. These sites pose particular challenges in terms of wound management.

Although EPT is well tolerated by patients, activation of the MedPulser may cause unpleasant “electric shock” sensations due to spasm of underlying muscles or due to pain relayed by local nerves that were stimulated by the MedPulser current. Our patient was treated under general anesthesia, although sedation and local anesthesia have been used in well accessible lesions, even in an outpatient setting [20].

In conclusion, electroporation therapy is a novel treatment potentially effective for sarcoma utilizing intralesional bleomycine. It is well tolerated and easy to perform in well accessible sites.
